# Ultra-Sensitive and Fast Humidity Sensors Based on Direct Laser-Scribed Graphene Oxide/Carbon Nanotubes Composites

**DOI:** 10.3390/nano13091473

**Published:** 2023-04-26

**Authors:** Ammar Al-Hamry, Tianqi Lu, Haoran Chen, Anurag Adiraju, Salem Nasraoui, Amina Brahem, Danica Bajuk-Bogdanović, Saddam Weheabby, Igor A. Pašti, Olfa Kanoun

**Affiliations:** 1 Measurement and Sensor Technology, Department of Electrical Engineering and Information Technology, Chemnitz University of Technology, 09107 Chemnitz, Germany; tianqi.lu@etit.tu-chemnitz.de (T.L.); adiraju.anurag@etit.tu-chemnitz.de (A.A.); salem.nasraoui@etit.tu-chemnitz.de (S.N.); amina.brahem@etit.tu-chemnitz.de (A.B.); saddam.weheabby@etit.tu-chemnitz.de (S.W.); 2 University of Belgrade—Faculty of Physical Chemistry, Studentski trg 12–16, 11158 Belgrade, Serbia; danabb@ffh.bg.ac.rs (D.B.-B.); igor@ffh.bg.ac.rs (I.A.P.)

**Keywords:** laser direct writing, reduced graphene oxide, carbon nanotubes, nanocomposite, humidity sensor, impedance spectroscopy

## Abstract

In this paper, the relative humidity sensor properties of graphene oxide (GO) and graphene oxide/multiwalled nanotubes (GO/MWNTs) composites have been investigated. Composite sensors were fabricated by direct laser scribing and characterized using UV-vis-NIR, Raman, Fourier transform infrared, and X-ray photoemission spectroscopies, electron scanning microscopy coupled with energy-dispersive X-ray analysis, and impedance spectroscopy (IS). These methods confirm the composite homogeneity and laser reduction of GO/MWNT with dominant GO characteristics, while ISresults analysis reveals the circuit model for rGO-GO-rGO structure and the effect of MWNT on the sensor properties. Although direct laser scribing of GO-based humidity sensor shows an outstanding response (|Δ*Z*|/|*Z*| up to 638,800%), a lack of stability and repeatability has been observed. GO/MWNT-based humidity sensors are more conductive than GO sensors and relatively less sensitive (|Δ*Z*|/|*Z*| = 163,000%). However, they are more stable in harsh humid conditions, repeatable, and reproducible even after several years of shelf-life. In addition, they have fast response/recovery times of 10.7 s and 9.3 s and an ultra-fast response time of 61 ms when abrupt humidification/dehumidification is applied by respiration. All carbon-based sensors’ overall properties confirm the advantage of introducing the GO/MWNT hybrid and laser direct writing to produce stable structures and sensors.

## 1. Introduction

Since the first humidity sensor was invented in the 15th century [1], humidity sensors are becoming increasingly important in everyday human life as well as domestic and industrial processes [2,3,4]. From the early days in fields, such as agriculture and weather forecasting, to the present-day electronic devices, paper industries, and medical sensors, the requirements for the sensitivity and response time of humidity sensors are constantly increasing [4]. However, most current commercial humidity sensors generally suffer from low sensitivity, especially in low and high humidity ranges, and slow response time, which increases the difficulty and cost of developing supporting circuits. In order to avoid this bottleneck, it is necessary to develop sensing materials with better sensor properties and a simple electronic interface where impedimetric sensors are the best to fulfill due to their simplicity, cost, and adaptability to different circuits and manufacturing types [4].

Generally, several principles of humidity measurement, such as impedance, capacitance, resistance, gravimetric (frequency), optics, fluorescence, and power generation/self-powered, utilize different kinds of conventional and novel materials, e.g., ceramics, polymers, and nanomaterials, as summarized in different reviews [2,3,4,5,6,7,8]. For example, impedimetric sensors comprise a substrate, an electrode, and a humidity-sensitive coating. Their characteristics are mainly based on the hygroscopic nature of the material and the structure of the electrode, which leads to the change in the impedance upon the change of relative humidity (RH). Gravimetric sensors, such as quartz crystal microbalance (QCM) [9] or surface acoustic wave (SAW) [10], measure the frequency change due to the effect of humidity. Several techniques for optical humidity sensors are also summarized in [2,6]. One of the main principles is the change of reflected optical signal due to the water molecules adsorbed on the surface of a sensing film. Considering that water molecules and sensing films have different refractive indices, changes in humidity affect the intensity/wavelength of the refracted light [11,12]. 

Based on the analysis of the different types of sensors, the ability of a material to interact with water molecules is an essential part of how sensitive it is to humidity. Thus, one area of research for humidity-sensitive materials is synthesizing materials better at absorbing water molecules or finding materials with a high water-molecule-adsorption capacity, hydrophilicity, response/recovery time, less hysteresis, time stability, etc. These are the bottlenecks for the development of humidity sensor materials. Recently, a wide spectrum of materials, material hybrids, and composites have been developed based on different kinds of principles such as paper-based [13], cellulose composites [14], metal oxides [15,16], nanoparticle [10], metal-organic framework [17]. Among widely studied materials for humidity measurement are graphene and its derivatives, especially graphene oxide (GO), using different sensor principles [5,18,19,20] with high sensitivity and fast response times. 

Graphene has attracted more attention due to its excellent electrical, mechanical, and chemical properties [21]. Unlike the highly conductive graphene, GO is an insulating to semi-metallic material, a graphene derivative containing epoxy, hydroxyl, and carboxyl groups covering the surface [22]. It has attractive mechanical, electronic, and chemical properties for application in humidity sensors. These functional groups containing oxygen serve as adsorption sites for water molecules in the air. The physisorbed water may be ionized under an electric field as the multilayer physical adsorption develops, producing a significant amount of ionized water, i.e., hydronium ions (H_3_O^+^) as charge carriers [23]. This causes a significant change in the impedance of the GO film. Reduced graphene oxide (rGO), fabricated by reducing GO by different methods, is a form of graphene with similar properties to graphene (large specific surface area). However, rGO generally has more defects and is of lesser quality than graphene made directly from graphite. rGO also contains residual oxygen and other heteroatoms. Therefore, the reduction process can be controlled as needed to control the number of oxygen-containing functional groups in rGO for specific applications via thermal, chemical, and optical techniques [24]. 

Many researchers have paid attention to GO and rGO due to their low cost and the possibility of large-scale production to fabricate high-performance humidity sensors [23,25,26,27,28,29,30,31,32,33,34]. For example, H. Bi et al. [23] reported an ultrahigh capacitive GO-based humidity sensor produced using drop-casting on silver interdigitated electrodes. The sensor has a response of 37,800% at RH 15–95%, response/recovery times of 10.5/41 s, and confirmed stability over 30 days. In comparison by the authors, the sensitivities of most conventional capacitive humidity sensors are only from 43% to 2900%. The response time of this humidity sensor was 1/120 to 1/4 of that of conventional humidity sensors. Additionally, S. Borini et al. [25] reported rGO as an ultra-fast humidity sensor with a response time of 30 ms where rGO films’ thickness significantly influences response time. For extremely thin GO films (nanoscale), the response time of rGO humidity sensor is faster than 1 s, which is among the best results in the literature. M. Tang et al. [33] as fast a response as 50 ms using supramolecular polymerization of GO and aniline as freestanding films. In addition, GO-based composites, such as poly(vinyl alcohol)-reinforced and toughened with poly(dopamine)-treated GO [26], graphene/carbon nanotubes [35] have been demonstrated with a rather limited range as humidity sensors. The addition of GO to metallic oxide was proven to improve the response and recovery times to achieve 0.2/1 s response /recovery times for Ho_2_O_3_/GO composites [36].

Combined with direct laser writing, GO structured sensors have the advantages of high performance, low cost, miniaturization, patterning, low-temperature operation, non-toxic, and no-photomasks [37]. Apart from that, laser-scribed rGO also shows suitable conductivity and chemical stability, allowing it to be utilized in humidity sensing [38,39]. Fast and highly sensitive graphene-based humidity sensors produced in a single-step laser fabrication have been reported [40,41,42,43] as films with all-graphene interdigitated rGO-GO-rGO structure in a wide humidity range of 6.3% to RH 100% and response/recovery in the range of several seconds, compared among the best performing materials. Moreover, laser-structured GO sensors also exhibit good repeatability and long-term stability (>one year) [41].

Because the interaction between the water molecules and GO oxygen functional groups is strong, GO-based humidity sensors exhibit significant hysteresis and inconsistent behavior [27]. In addition, their sensing properties are often described by slow response and recovery times [26]. Furthermore, because of the high impedance of GO (conductivity often below 1 × 10^−4^ S/m) [44], high potentials are needed to be applied to obtain measurable currents, leading to greater power consumption [23,33]. 

Carbon nanotubes (CNTs), materials with intrinsic mechanical and electronic properties, can improve composites’ mechanical and electrical conductivity and are often used as reinforcing agents for composite materials [45]. CNTs are insoluble in water, but GO is dispersible due to oxygen functional groups [46]. Therefore, CNTs are dispersed between the GO layers during a straightforward solution assembly to create graphene-CNT hybrids as cheap, substrate-compatible, and simple techniques [47,48]. Adding multiwalled CNTs (MWNTs) to GO dispersion can effectively maintain the long-term stability of the MWNTs/GO dispersion and prevent GO aggregation during reduction due to the π–π interaction between the sidewalls of the MWNT and the rGO hydrophobic region [49]. This combination of GO and CNTs has a synergistic impact that improves the electrical conductivity during the reduction and electrocatalytic activity [50]. Nevertheless, the GO-CNT hybrids water aqueous behavior is an important subject that is yet to be investigated in detail, especially its long-term use and stability, e.g., decreased solubility and increased hydrophobicity after reduction [49]. GO/CNTs-based sensor shows different sensitivities by the effect of GO flake swelling and thus has intertube effect sensitivity doubling the sensitivity and enhancing the stability of CNT-based sensor [51], and lowering hysteresis [52,53]. 

In this paper, we investigate GO and GO/MWNT composites reduced and structured by laser scribing. rGO/MWNT-GO/MWNT-rGO/MWNT structure is proposed where rGO/MWNT works as interdigitated electrodes and GO/MWNT works as a water vapor-sensitive layer. The sensitivity toward humidity is compared to different structured sensors. The repeatability, response time, and long-term stability have been investigated. Compared to many literature sources discussing humidity sensors based on GO, highly sensitive GO-based sensors can be obtained and further improved by GO/CNT composite to obtain all carbon-based humidity sensors. It has several advantages over GO-based sensors. In addition, ultra violet-visible near-infrared, Raman, Fourier transform infrared, and X-ray photoemission spectroscopies, and electron scanning microscopy coupled with energy-dispersive X-ray analysis have been utilized to investigate the physical characterization of the composites and laser-based structures. Impedance spectroscopy measurement investigation was also carried out to explore the equivalent circuit model for the laser direct written sensors.

## 2. Materials and Methods

### 2.1. Materials and Preparation

**Figure 1:**
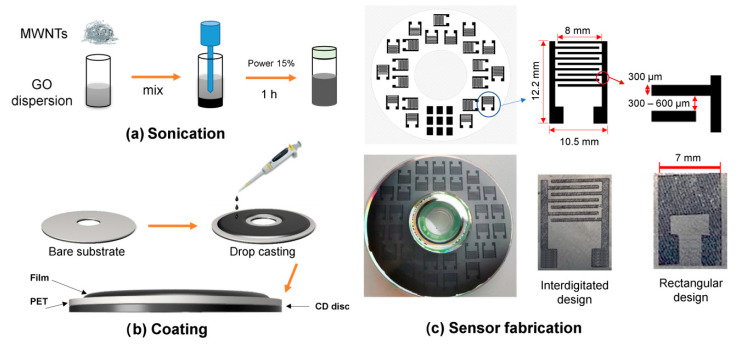
(**a**) GO/MWNT composite preparation, (**b**) preparation of the coating for laser scribing and (**c**) digital design and realized sensor after scribing.

GO aqueous dispersion (0.4 wt.%) was purchased from Graphenea Inc., San Sebastián, Spain. The GO dispersion was diluted with deionized water to 0.3 wt.% GO. A total of 12 mL of 0.3 wt.% GO solution was taken out for GO humidity sensors. A total of 12 mL of 0.3 wt.% GO solution was used for the preparation of GO/MWNT dispersion. A total of 2.4 mg of MWNT powder (CAS 308068-56-6, Sigma-Aldrich, St. Louis, MO, USA) was added to the GO solution. The solution was placed for sonication and treated at 30% power for 45 min using a Bandelin sonication device (200 W) (BANDELIN electronic GmbH & Co. KG, Berlin, Germany) to disperse the CNTs in GO. Thus, a 12 mL MWNT/GO (GO/MWNT) solution was obtained, having a mass fraction of MWNT of 0.02 wt.% and a mass fraction of GO of 0.3 wt.%, Figure 1a. 

For the preparation of the sensors based on DVD laser scriber, every solution was drop-casted on an area of 90 cm^2^, which is the area available for scribing by Lighscribe DVD disc covered by polyethylene terephthalate (PET) substrate, Figure 1b. Based on the volume and concentration of the GO dispersion (12 mL, 0.3 wt.%), the GO loading was 0.4 mg per cm^2^. To ensure the uniformity of the film, drop coating and drying processes were carried out in a compartment with a self-balanced workbench. After 24 h of drying process at room temperature, discs with GO and GO/MWNT films were obtained. The digital design as a rectangular film/interdigitated electrode (IDE) was made by laser scribing software (Nero CoverDesigner, Nero AG., Karlsruhe, Germany). The DVD laser with a wavelength of 785 nm was used to directly write the electrodes. The laser reduction was based on DVD driver (HP DVD AD-7721H) with 5 mW power focused on a spot size of 50 µm [54]. In addition, the process of writing was repeated six times to obtain a complete reduction of the patterned areas. Two sensor structures were realized; one as a rectangle of fully reduced GO (7× 5 mm) and the second as an interdigitated structure (rGO-GO-rGO) having the dimension 10.5 × 12.2 mm as shown in Figure 1c. For IDE sensors, the finger width is 300 µm, and the gap between fingers was changed between 300 and 600 µm. For most of the tests, the gap distance of 600 µm was taken because of its reproducibility, Figure 1c. More details about IDE sensor dimensions and their microscope optical images are shown in Table S1 and Figure S1. The silver paste was used to connect wires to the sensor terminals. This was applied for GO/MWNT to have rGO/MWNT resistive sensor with a rectangular shape (marked hereafter simple as rGO/MWNT) as well as (rGO/MWNT-GO/MWNT-rGO/MWNT) IDEs. The latter types of sensors obtained by making IDE structures are denoted hereafter as GO IDE and GO/MWNT IDE (see Figure 1c).

### 2.2. Physical Characterization

The optical absorption properties of the deposited GO and GO/MWNT dispersions were investigated by UV-vis-NIR measurements using a Cary 60 spectrophotometer (Agilent, Santa Clara, CA, USA). Raman spectra, excited with a diode-pumped solid-state high brightness laser (excitation wavelength 532 nm), were collected on a DXR Raman microscope (ThermoScientific, Waltham, MA, USA) equipped with an Olympus optical microscope and a CCD detector. The laser beam was focused on the sample using an objective magnification of 10×. The scattered light was analyzed by the spectrograph with 900 lines mm^−1^ grating. Laser power on the sample was kept at 1 mW to prevent thermal degradation/reduction of the samples. FTIR measurements were performed using spectrometer INVENIO S (Bruker, Leipzig, Germany). All the measurements were recorded in attenuation total reflection (ATR) mode on Germanium crystal from 550 to 4000 cm^−1^. The baseline corrections and compensation of peaks due to the atmosphere were performed in OPUS software provided with the instrument.

SEM-EDX characterization was performed using Phenom ProX scanning electron microscope (Phenom, Eindhoven, The Netherlands). Before the analysis, samples were coated with a thin Cu layer to improve the visibility of carbon structures. 

XPS was performed to investigate the chemical speciation on the GO based sample’s surfaces. SPECS Systems with XP50M X-ray source for Focus 500 and PHOIBOS 100/150 analyzer were used to evaluate the samples. Al Kα source (1486.74 eV) at 12.5 kV and 32 mA was used for this study. Survey spectra (0–1000 eV binding energy) were recorded with a constant pass energy of 40 eV, step size 0.5 eV, and dwell time of 0.2 s in the FAT mode. High-resolution spectra of O 1s and C 1s peaks were recorded with a constant pass energy of 20 eV, step size of 0.1 eV, and dwell time of 2 s in the FAT mode. Spectra were obtained at a pressure of 9 × 10^−9^ mbar. SPECS FG15/40 electron flood gun was used for charge neutralization to minimize the effects of charging at the samples. All the peak positions were referenced to C1s at 284.8 eV. Spectra were acquired using the manufacturer’s SpecsLab data analysis software and processed using the commercial CasaXPS software suite.

### 2.3. Humidity Characterization

For the acquisition of impedance spectra, saturated salt fixed humidity points were used. The used salts (LiCl, MgCl_2_, Mg(NO_3_)_2_, NaCl, KCl, K_2_SO_4_) were reagent grade with at least 99% purity (Sigma-Aldrich, Taufkirchen, Germany). The fixed points provided by the binary aqueous solutions were checked by a calibrated sensor and amounted to 11.3%, 32.78%, 52.89%, 75.29%, 84.34%, and 97.3% relative humidity. An impedance analyzer 4294A (Keysight, Santa Rosa, CA, USA) was used to measure the impedance spectra in the range 40 Hz–110 MHz, as shown in Figure S2a. This setup was also used to carry out the stability test. For the stability test, 5 sensors of each material were fabricated and put at each humidity point for the duration of the test. For other measurements (calibration and repeatability tests) two gas flow humidity measurement setup was used where two mass flow controller FLOW BUS (Bronkhorst, Veenendaal, The Netherlands) were utilized to adjust the humidity inside a chamber with a target sensor, Figure S2b. A temperature/humidity SH85 calibrated sensor was used as a reference connected to an Arduino UNO. LabView program was used to control all devices. In these experiments, Agilent LCR Meter 4284A (Keysight, Santa Rosa, CA, USA) was used to measure the impedance, while the range of humidities was 10–90% RH. The humidity and temperature profiles of the humidity measurement chamber are presented in Figure S2c. For the repeatability test, alternating values of 10% and 80% RH were used when proving the stability of the sensor over time and for the test of response/recovery time. 

## 3. Results and Discussion

### 3.1. Physical Characterization 

**Figure 2:**
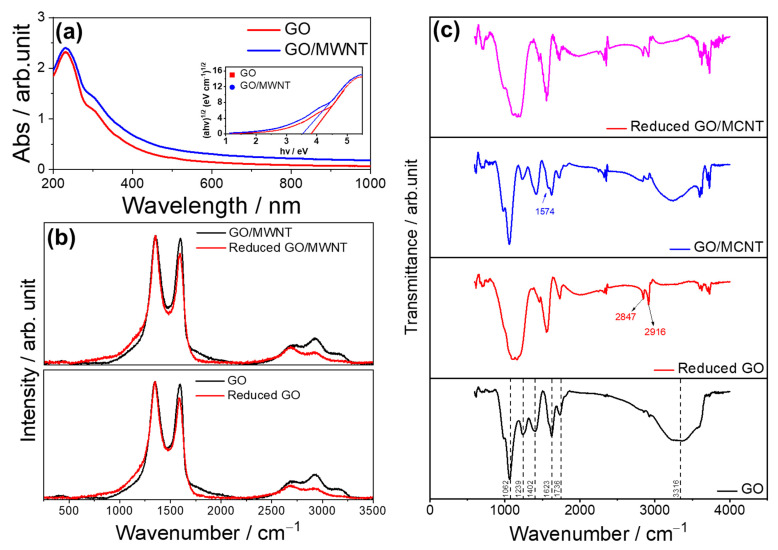
Optical spectroscopy measurement (**a**) UV-vis-NIR of GO and GO/MWNT dispersions (inset is Tauc plot), (**b**) Raman spectroscopy, and (**c**) FTIR of un- and reduced films of GO and GO/MWNT.

The UV-vis spectra of the GO and GO/MWNT dispersions (see Figure 2a) were collected after the dispersions were diluted 50 times in water. The absorption peak in the GO dispersion spectra at around 230 nm indicates the π–π* transition of the C-C bond. In addition, the shoulder at 300 nm, which indicates the n–π* plasmonic transition of C=O bonds, was noticed [55]. The GO absorption peak at 230 nm decreases as the GO concentration decreases in GO/MWNT dispersion. In the inset of Figure 2a, the optical band gap energies were determined using the Tauc plots extracted from the UV-vis-NIR spectra according to [56]. The band gaps decrease from 3.80 for pristine GO to 3.52 eV for GO/MWNT. These results confirm the role of MWNT contribution to lowering the composites’ overall bandgap, which will increase the conductivity. 

Raman spectra of GO and GO/MWNT composites (Figure 2b) show high similarity, indicating that the GO component dominates the spectra. Such a similarity is maintained after reduction, suggesting that now rGO determines the overall spectral response. In both cases, relative ratios of D and G decrease after reduction, and the intensity of the G band, found around 1590 cm^−1^, reduces. In addition, the changes are visible in the 2D region above 2500 cm^−1^, confirming effective reduction. This conclusion is in agreement with previous reports [39,54]. The change of color from brownish to black after reduction is observable in optical and microscope images in Figure 1 and Figure S1. The impedance measurement confirms the effective reduction, as will be discussed in the next sections. 

FTIR spectra were implemented for qualitative confirmation of the functional groups on the reduced and unreduced surfaces of GO and GO /MWNT composite. As can be observed from Figure 2c, the FTIR spectra of GO have bands at around 1062, 1239, 1623, 1736, and 3316 cm^−1^, which can be assigned to C–O–C, C–O, C=C, C=O, O–H stretching vibrations, respectively [49]. The band at 1402 cm^−1^ arises due to the bending vibrations of O–H in the carboxylic group [57]. After the addition of MWNTs in the GO matrix, the intensity of all the bands reduced and shifted considerably compared to the GO spectra, which are in accordance with previous studies [58,59]. In addition, a small shoulder peak at around 1574 cm^−1^ is formed, which can be related to the π–π interactions between MWNTs and GO [60]. 

After the laser irradiation, the bands related to -OH functional groups at 3316 cm^−1^ are entirely diminished or reduced (1239, 1736 cm^−1^) for both rGO and rGO/MWNT composite, as seen in the spectra, confirming the reduction of GO [61,62]. Nevertheless, not all the oxygen groups are reduced, and the residues are still present on the surface. However, the residuals do not create much impact on the electrical properties of the reduced surface. Furthermore, two new bands at 2847 and 2916 cm^−1^ are observed for the reduced films, which can be attributed to symmetric(sCH_2_) and asymmetric stretching (aCH_2_) of the C-H bonds in the lattice [63,64].

**Figure 3:**
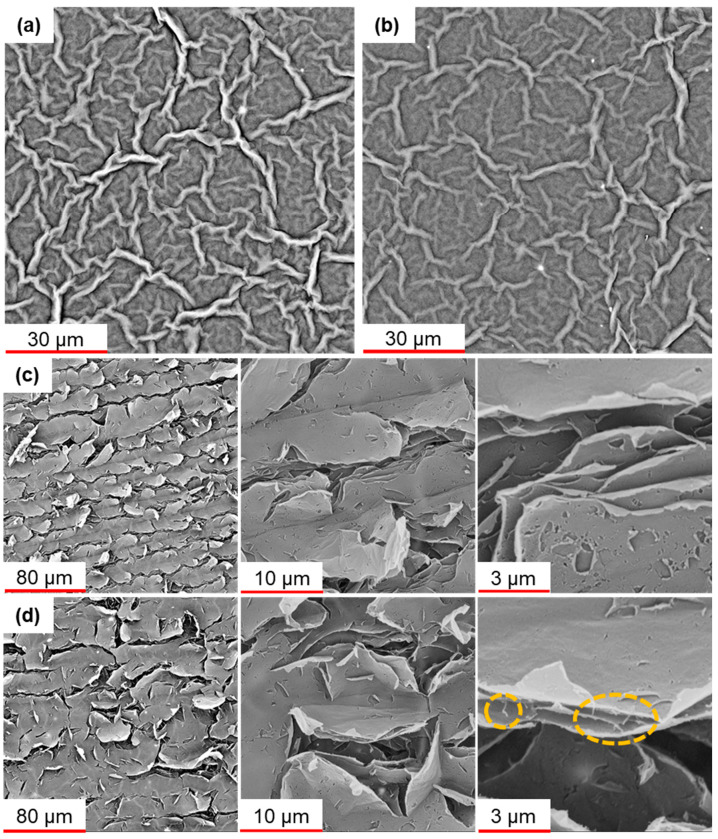
SEM images of (**a**) unreduced GO, (**b**) unreduced GO/MWNT, (**c**) laser-scribed rGO at different scales, and (**d**) laser-scribed reduced GO/MWNT at different scales where the CNT can be observed in the image with a scale bar of 3 μm.

**Figure 4:**
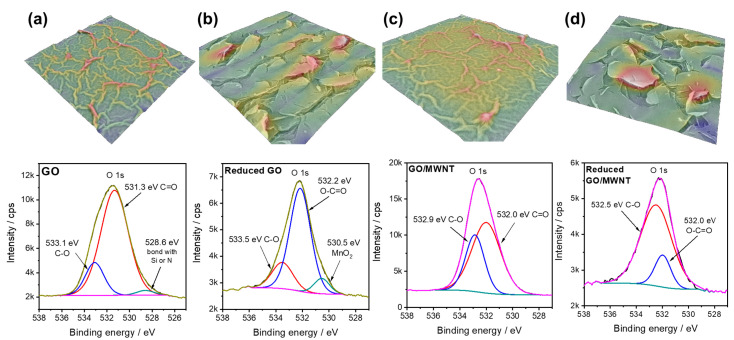
3D SEM surface reconstruction and high-resolution O 1 s spectra of (**a**) GO, (**b**) reduced GO, (**c**) GO/MWNT composites, and (**d**) reduced GO/MWNT composite.

SEM analysis of GO and GO/MWNTs composites showed similar morphologies before reduction. The layers are relatively smooth, with wrinkle networks that likely formed during the drying process (Figure 3) and roughness factor (determined by 3D SEM reconstruction) close to 7 for both layers (evaluated as real surface area divided by geometric cross-section). After the reduction step, the morphology changes, with numerous cracks appearing on the surface, going deep into the layers. The determination of the roughness factor is difficult in this case, as SEM cannot sample deeper regions of the sensing layers. The overall morphology of the reduced GO and GO/MWNT composite is similar, but the reduced GO surface appears more cracked. In the GO/MWNT composite case, some nanotubes can be observed (Figure 3), but they are generally well-mixed with GO and integrated into the laminar structure of the composite layers. Such integration of GO and MWCNTs is likely the reason why the surface of the reduced GO/MWCNT composite is less cracked compared to the reduced GO layer. Overall, surface 3D reconstruction shows that surface roughness profiles (Figure 4) for all the studied layers are below 1 µm, while the cracks in the layer cause more pronounced roughness. 

The chemical composition of the studied sensing layers was investigated using EDX and XPS. According to EDX analysis, C and O are uniformly distributed in all the samples (Figure S3, Supplementary Information), while some elements were also found in traces. These include S and N, but also Mn, which likely originates from the synthesis of GO. Upon the reduction of the surface, XPS survey spectra showed that the C:O ratio in GO increased from 2.56 to 13.2. The same was observed for GO/MWNT composite. Before the reduction, the C:O ratio was 2.62, and after the reduction, it was 16.82. The character of the surface oxygen functional groups also changed, in line with the result of FTIR analysis. However, single C-O, carbonyl, and carboxylic groups were found in all the cases. However, we note that the effect of the laser reduction is limited to the surface layers, as can be concluded from the comparison of C:O ratios obtained by XPS and EDX. As already mentioned, XPS, which is a surface-sensitive technique, showed a high increase in the C:O ratio ratios upon the reduction. The same trend is observed by using EDX, which samples deeper surface layers. In the case of EDX, for GO, the C:O ratio increased from 1.2 (non-reduced) to 2.3 (reduced film). In the case of GO/MWNTs composite, the C:O ratio increased upon reduction from 1.3 to 1.9. Overall, the obtained results suggest an efficient reduction of the GO component and its conversion to reduced GO, which increases the conductivity of the electrical contacts (Figure 1c). However, we believe a gradient of the GO reduction state exists from the surface to deeper layers of the reduced films, where the highly reduced GO is present on the surface, while in deeper layers, the reduction degree is lower. 

### 3.2. Humidity Dependence Characterization

#### 3.2.1. Impedance and Capacitance Behavior of Laser-Structured Sensors

To investigate the moisture-sensitive properties of GO materials, impedance measurements were performed under different humidity environments provided by the humidity-fixed points of binary saturated aqueous solutions. Four sensors based on GO and GO/MWNT have been investigated. They are the laser-scribed rectangular shape (rGO and rGO/MWNT sensors) and the interdigitated (IDE) structures having (rGO-GO-rGO and rGO/MWNT-GO/MWNT-rGO/MWNT forms), abbreviated as GO IDE and GO/MWNT IDE sensors, as shown in Figure 1c. 

**Figure 5:**
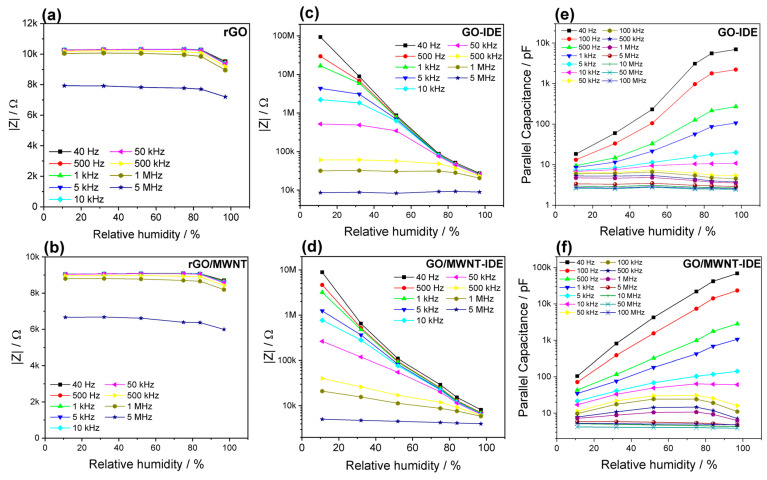
Humidity dependence of the impedance for (**a**) rGO, (**b**) rGO/MWNT, (**c**) GO-rGO IDE, and (**d**) rGO/MWNT-GO/MWNT IDE sensors. Humidity sensitivity of the capacitance for (**e**) GO and (**f**) GO/MWNT IDE sensors.

To evaluate the sensitivity to relative humidity, the impedance modulus is plotted against relative humidity at different frequencies, Figure 5. For fully laser-reduced sensors, rGO and rGO/MWNT (Figure 5a,b), the humidity dependence is almost unchanged for most of the test range, 11–75%. A slight increase in the impedance can be noticed, which attributes to swelling where water gets within the layers and can lead to an increase in the interlayer distance [65,66]. However, several factors can affect while adsorption of water molecules, such as oxygen group dissociation, hydrophobicity, the donor property, and the p-type nature of rGO [18,42,67]. From the slope of the curve, the slight increase can be estimated to be 0.006%/RH% to the initial value. After 97% RH, a relatively considerable drop occurs, i.e., 7%, of the impedance. rGO/MWNT film has a smaller impedance than rGO films. However, both have similar humidity dependence. The impedance increases slightly in the range of 11–75% with 0.007%/RH% and has a decline at 97% RH of 4%, which may be attributed to the GO materials remaining unreduced in the film, which contributes inversely to rGO/CNTs [35] and causes the lowering of impedance at high humidity. This can be noticed in Bode plots (Figure S4) where slight changes of impedance and phase are observed versus relative humidity over a wide frequency range. In previous work [65], rGO humidity dependence was found to be a function of the degree of reduction temperature and has a notable change at high humidity value. It can be concluded that highly laser-reduced GO can have insensitive humidity properties and hence does not necessitate using a passivation layer in a wide range of humidities. This insensitivity property was utilized by [38] to obtain a temperature sensor. However, encapsulation was still necessary to avoid the influence of high humidities.

For IDE sensors, Figure 5c,d evaluate impedance amplitude at different frequencies; the humidity dependence can be well seen on a logarithmic scale. The impedance drops by 4 orders of magnitudes (from several tens of MΩ to about 27 kΩ at 40 Hz) in the range of 11–97% for the rGO-GO-rGO IDE electrode structure. The sensitivity for GO/MWNT IDE sensor is less, knowing that the initial resistance is in the range of several MΩ and decreases to less than 10 kΩ, which indicates lesser sensitivity than GO by one order of magnitude. In both cases, there is a significant dependence on the frequency. For GO-based sensors, the humidity response range is reduced when the test frequency is higher than 5 kHz. Overlap observed in the real part of the impedance in the Bode plot results in non-monotonic humidity dependence behavior, Figure S5a. In comparison, GO/MWNT sensors have excellent sensitivity in full humidity and wide frequency ranges (up to 1 MHz). Frequency dependency causes a decrease in the sensitivity of GO sensors, either capacitive [68] or impedance sensors, and hence shrinks the operation range, which is the case also in different works, as in [69]. A flat response at high frequencies is observed for impedance sensors (Figure 5c) at low humidities because the water molecule cannot follow the electrical field, and for capacitive sensors, the sensitivity decreases tremendously [29]. 

To highlight the reasons for differences in sensitivity at low humidity, on the one hand, rGO rectangle shape sensors, as stated above, have behavior similar to a large extent to the behavior of carbon nanotubes and graphene, where the increase in humidity results in the rise of resistance. They are highly reduced GO and GO/MWNT as the initial resistance at 11% RH is very low. This means they are of hydrophobic nature. So, at low humidity, water molecules are only adsorbed on the surface and as having donating properties, while the rGO and MWNT films are of p-type nature, and thus the resistance increases due to the increase in holes [70]. As the structure is multilayer, there is a possibility for the water molecules to form a conductive percolation chain as the humidity increases [30], causing a drop in the impedance at higher humidity. Both factors play against each other to lower the sensitivity. On the other hand, GO is highly hydrophilic, and film formation of conductive chains may occur at lower humidities with a dispersion-like mechanism (straight line in the complex impedance at low-frequency range) such as in [71,72]. Additionally, at this low RH%, the water molecules are chemisorbed and physisorbed at hydrophilic and vacancy sites by hydrogen bonding, and charges can hop between neighboring hydroxyl groups linked by water molecules. This leads to a reduction of the impedance significantly, even at low RH%.

The capacitance measurements versus humidity at different frequencies are plotted in Figure 5e,f. GO/MWNT outperforms the GO sensor as a capacitive sensor. The initial capacitance of GO and GO/MWNT are 18 pF and 104 pF (11 RH% and 40 Hz). The dielectric properties of the GO are improved by the addition of MWNT, where the capacitance can reach ~68.9 nF at 97 RH% while it is 6.9 nF for the GO sensor. In addition, rGO/MWNT has suitable linearity (in a semi-log scale). Thus, the overall sensitivity of GO/MWNT is 798 pF/%RH. However, the capacitance characteristics decline fast with the frequency effect. Having a bigger initial capacitance, the characteristic peak frequency of MWNT is higher than GO as noticed in impedance imaginary Bode plots (Figure S5), which makes it usable as an impedance humidity sensor for a wide frequency range.

To summarize, MWNT reduces the high impedance of GO material at a low-humidity range, and thus, the conductivity of GO/MWNTs materials has been significantly improved below RH 50%. Under normal indoor conditions, the rGO/MWNT sensor impedance is only 16% of the GO sensor at 1 kHz. MWNTs, fill the gaps between GO material by connecting independent 2D GO films to form a 3D structure, which further increases the specific surface area of the material and makes the charge transport between GO and/or rGO flakes more effective [50,73]. It helps to adsorb water molecules in the humid air better as it increases the interlayer distance and thus provides an accessible surface [73]. The more water molecules the GO material adsorbs, the bigger the conductivity of the material. With the continuous increase in humidity, the water molecules adsorbed by GO and GO/MWNT are gradually approaching the saturated state, so the electrical conductivity gradually approaches the same level corresponding to the formation of water films. For both sensors, the active area between reduced electrode fingers is rich with oxygen groups from GO, which plays an essential role in the humidity sensitivity mechanism. 

#### 3.2.2. Sensor Properties of Laser-Structured IDE Sensors

**Figure 6:**
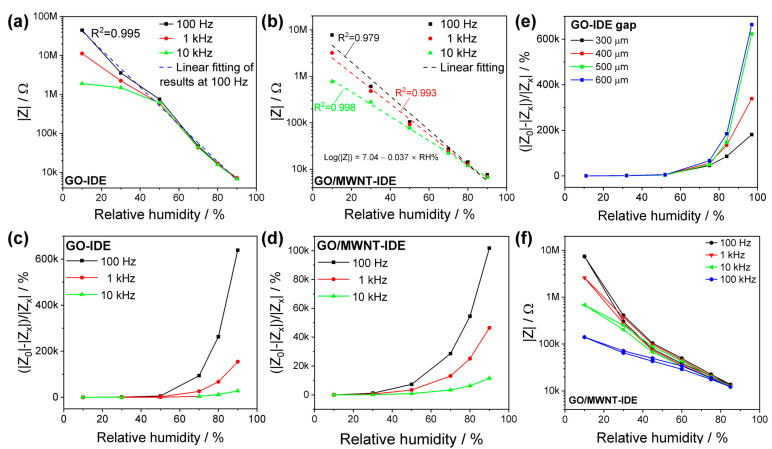
Relative change of impedance modulus versus relative humidity of (**a**) GO and (**b**) GO/MWNT laser-scribed IDE sensors, (**c**) geometry effect on the sensitivity, and (**d**) hysteresis test for GO/MWNT IDE, (**e**) impedance response with the variation of the gap between rGO electrode fingers, and (**f**) hysteresis of rGO/MWNT IDE sensors.

To test the humidity sensor and construct calibration curves for the developed sensors, the humidity chamber base on two gas flows was used to obtain the GO and GO/MWNT IDE sensors’ properties. Given the link between absolute humidity, relative humidity, and temperature, the recorded temperature was kept constant at 23 °C to ensure the accuracy of the measurements. The results are shown in Figure 6a,b for impedance modulus versus relative humidity. According to the characteristic that the sensor impedance decreases with increasing humidity, the following formula is used to calculate the response as the relative change of impedance modulus.

(1) $$\mathrm{Response}\;R=\frac{\Delta Z}{Z}\%=\frac{Z_{0}-Z_{x}}{Z_{x}}\cdot 100\;\%$$

*Z*_0_ is the impedance of the humidity sensor at RH 10%, and *Z_x_* is the impedance of the humidity sensor at a particular RH. The results are shown in Figure 6c,d. The sensitivity of the GO IDE humidity sensor is much higher than that of the GO/MWNT IDE. The greatest relative change of the GO IDE sensor is 6.30 × 10^5^% at 97% (100 Hz). However, for GO/MWNT, the maximum relative change is 1.63 × 10^5^% (100 Hz) with better linearity in the semi-log scale, and impedance ranges from 7.3 kΩ to 7.7 MΩ. The high impedance of GO IDE will increase the difficulty of subsequent sensor circuit design and increase power consumption [5]. In addition, as the frequency increases, the sensor characteristics tend to be less sensitive in the low-humidity range, which can be seen typically for different high-ohmic GO-based sensors [29]. 

It can be concluded that two factors control the overall sensitivity between the lowest/highest humidities. The first is the initial impedance of the unreduced GO-based sensitive area, and the second is the final resistance induced by proton hopping, in addition to the rGO fingers’ impedance. It is noticed that the final resistance is almost in the same order of magnitude for both materials GO and GO/MWNT. The resistance of the water film itself depends on the geometrical shape [42] and gap of IDE structure (as in Figure 6e for GO sensor for sensors described in Figure S1) as well as the hydrophilic and surface defect properties of the materials, which originate from preparation parameters, thickness and morphology [28,49,67,74].

Based on the discussion so far, we choose mainly GO/MWNT for subsequent sensor characteristics and at a test frequency of 1 kHz.

The hysteresis test result of the humidity sensor is shown in Figure 6f at different frequencies 100 Hz, 1 kHz, 10 kHz, and 100 kHz. The hysteresis was calculated, e.g., at the frequency of 1 kHz, to have a maximum value of 3.1% at 60% RH, according to Equation (2) [3].

(2) $$H=\frac{\left|Z_{D}\right|-|Z_{A}|}{S}$$

*Z_D_* and *Z_A_* are the impedances modulus measured during desorption and adsorption, and *S* is the sensitivity calculated based on Equation (3), where |*Z_x_*| and |*Z_y_*| are the impedance modulus obtained at humidity points *RH_x_*% and *RH_y_*%.

(3) $$S=\frac{\left|Z_{x}\right|-|Z_{y}|}{RH_{x}-RH_{y}}$$

#### 3.2.3. Stability and Repeatability Tests under Humid Environments

**Figure 7:**
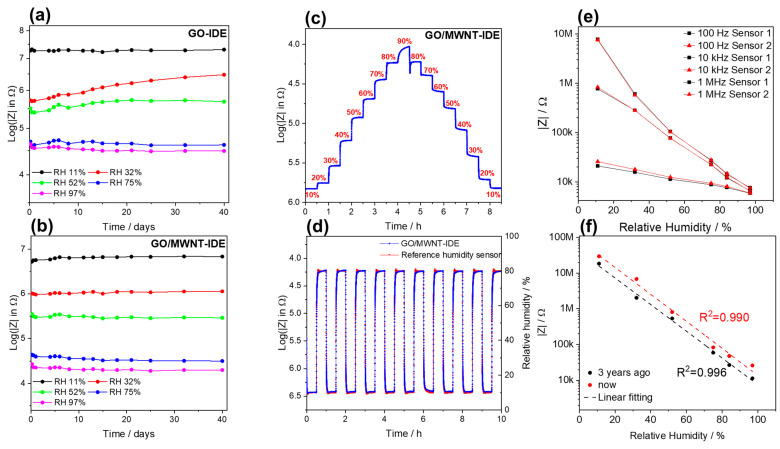
Stability of the (**a**) GO and (**b**) GO/MWNT IDE sensors inside different humidity chambers, (**c**) Step response 10%-90%-10% versus time for GO/MWNT IDE sensor, (**d**) stability measurement over 10 h by cycling the humidity between 10% and 80%, (**e**) reproducibility of the characteristics by two different sensors, and (**f**) stable and producible sensitivity after 3-year interval.

In this section, the developed structured sensors were put in different humidities for a long time to study the stability of the sensors. Due to the long experimental period of the stability test, saturated salt solutions were used for this test. Five saturated salt solutions with five humidities, i.e., 11, 32, 52, 75, and 97%, were placed in sealed bottles (at ambient room temperature ~23 °C), and in each solution, four different sensors were put, i.e., rGO and rGO/MWNT films, and the IDE sensors (five each). The IDE GO and GO/MWNT-based sensors showed excellent stability in the humidity range of 11–97%, Figure 7a,b. However, GO/MWNT material was found to have better long-term stability than GO sensors. This could be attributed to MWNTs strengthening the GO film’s mechanical properties and water absorption behavior as a compromise of the hydrophilic-hydrophobic nature tuned by laser, making the sensor structure more robust. For the GO IDE sensor, in the 32–52 RH% range, an increase over time is notable, where swelling of flakes is more dominant, and it seems that more water molecules diffuse between flakes over time. Moreover, a non-monotonous behavior of impedance at this range is noticed clearly [30], which is frequency dependent. This is obvious because the highest values of hysteresis are found in this range by simply looking at the sensor impedance humidity characteristic reported in the literature. 

In addition, Figure S6a,b show the stability results for 40 days of testing period for those sensors. Reduced films show suitable stability and constant values of impedance. The difference in impedance comes from the deviation of the initial impedance of each of the five sensors of each material. This proves that rGO and rGO/MWNT materials can withstand different humidities and remain unchanged over a long period. 

Following that, the repeatability of the GO/MWNT IDE sensor was examined, where the step response procedure was carried out by measuring the sensor impedance over time for 8 h following the humidity profile inside the chamber. A staircase-wise profile from 10 to 90%RH and back to 10% at an almost constant temperature of ~22.4 °C was applied, as shown in Figure S2c. Figure 7c shows the staircase change of the sensor impedance (log scale, which indicates suitable sensor behavior). In Figure 7d, pulses of humidity alternating between 10% and 80% RH are applied to the sensor. The sensor shows excellent repeatability and recoverability following humidity set points, and thus it has excellent performance. In addition, the results of two different sensors are shown in Figure 7e, which have almost identical characteristics. Moreover, by comparing the sensor performance after 3 years interval in Figure 7f, it shows maintained sensitivity. There is a shift in the impedance values, which means calibration is needed over time.

#### 3.2.4. Response Time Test

**Figure 8:**
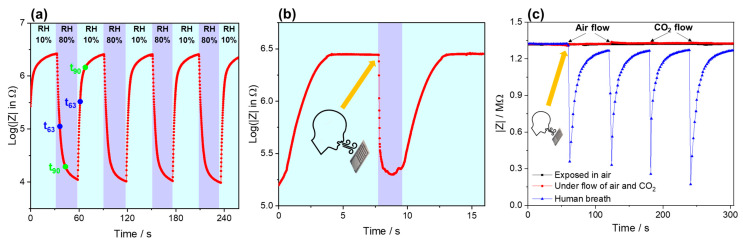
Investigation results of GO/MWNT-based humidity sensor showing the response/recovery times using (**a**) alternating humid and dry air using valves and (**b**) applied breath exhalation and dry air. (**c**) Comparison of the reaction by human breath, stable ambient air, air flow, and CO_2_ flow.

To test the humidity response and recovery time of the sensor, humidifying processes (wet air) and drying processes (dry air) were performed in a small chamber controlled by valves where humidifying will continuously inject highly humid nitrogen into the surface of the sensors under a constant flow speed to have a sudden increase in humidity. To ensure the humidity’s stability on the sensor’s surface, we fixed the materials/sensors very close (5 mm) to the gas supply port. After a preset time, the drying valve was opened, and the drying process was applied using dry nitrogen. Therefore, the humidity on the surface of the sensor is supposed to drop immediately from 80% to 10% and remain at 10%. By applying the procedure, the measured impedance at 1 kHz is plotted versus time (Figure 8a). The response time obtained by this procedure was determined as t_63_ and t_90_ and amounted to 4.1 s and 10.7 s, respectively. The recovery times, t_37_ and t_10_, were 3.0 s and 9.3 s, respectively. In order to have a more abrupt change, breathing (sudden and long exhalation) was applied, Figure 8b, followed by drying using dry clean air. The obtained response time to highly humid air caused by breath was 61 ms, and the recovery time was 2.3 s. By doing so, the applied change of humidity and change of resistance obtained by DC voltameter (logging rate of 10 ms), more accurate sensor properties are obtained. To make sure that the sensitivity is because of humidity, the sensor was tested under clean air and CO_2_ gas, where the effect of humidity is pronounced, Figure 8c. In comparison to the literature, especially those works based on GO, there are discrepancies in the reported response and recovery times, as seen in Table 1. Only work by Borini et al. [25] concluded the effect of thickness as a crucial factor. It may also be attributed to the initial properties of GO materials obtained by different routes and having different sizes in the micro and nanoscale. However, a systematic study may be needed to determine playing factors on the absorption and desorption reaction time.

The obtained results provide a suitable picture of the sensor characteristics indicating its suitable performance over many recent materials for humidity sensors. In comparison with many papers, high sensitivities are demonstrated (Table 1) with proven stability and repeatability, while some of the properties are not highlighted by previous works. Specifically, capacitive-based GO sensors, although high sensitivity was shown where the range of capacitance changes from several pF to µF on a limited low-frequency range. GO-based sensors are not purely capacitive as it has high lossy capacitance; at high RH, the resistive part is very small compared to that at the low RH% as shown in the impedance spectra. Measurement of capacitance is tricky if the cables are not considered as their stray capacitance can be dominant and, thus, electronics design limitation [2]. In addition, using an LCR meter for most capacitive sensors depends on the choice to measure Cp-Rp or Cs-Rs mode, which makes it tricky to design a capacitance measurement circuit for a wider range.

In view of several works, GO-based humidity sensors have been utilized to detect water vapor from different biological exertion, such as exhalation, skin humidity, and no-contact sensing [75,76,77]. Out of that, several actuators were demonstrated, e.g., to follow breathing rate, finger proximity, and keyboards to activate some processes. In such a manner, we demonstrate the use of the humidity sensor to switch on-off LED where the intensity of light can reflect the water absorption by the sensor when breathing or detecting the proximity of a human finger or wet stick. The results can be found in Video S1 in Supplementary Data. 

**Table 1:** Comparison of humidity sensing properties for GO and composites.

Sensor Material	Range (RH%)	Response	Sensitivity	Operation Frequency	Response/Recovery Time (s)	Hysteresis	Tested Stability	Ref
Pyranine-rGO	11–95	*Z_L_*/*Z_H_*: 6 × 10^5^	-	100 Hz	<2	-	-	[29]
G-carbon ink	25–95	-	12.4 Ω/RH%	DC	4/6	-	4 months	[34]
LrGO * tattoo	20–92	-	-	DC	30	-	-	[40]
GO on LIG *	0–97	ΔC/C%: 1825 × 10^3^	1825 pF/RH%	500 Hz	16/9	3.03%	-	[42]
LrGO	7–97	-	1.67 MΩ/RH%	50 Hz	-	-	-	[43]
LrGO	6.3–100	Voltage: 142.5	-	0.04 Hz	1.9/3.9		1 year	[41]
Syringe printing of SWNT-GO	30–90	ΔR/R%: 30	-	DC	-	-	4 months	[51]
LrGO	19–97.7		4770 pF/% RH	50 Hz	30/7	0.49%	30 days	[71]
GO/MWNT	11–97	-	7980 pF/% RH	-	5/2.5	-	-	[73]
GO on LIG	11–97	-	9150 pF/RH%	500 kHz	2	-	3000 cycles	[78]
GO/BP	11–97	ΔC/C%: 4.45 × 10^4^	-	10 Hz	GO: 2.7/4.6BP: 4.7/3.0	-	-	[79]
TiO_2_/CNC *	11–95	R/R0: 4.5 × 10^4^			22/13		40 days	[80]
rGO/PDMS	10–95	-	-		2.4/1.7	3%	-	[72]
HNTs	0–91.5	10^5^	-		0.7/57.5	4.7%	28 days	[81]
(In + Nb) co-doped HfO_2_ ceramics	11–94	*Z_L_*/*Z_H_*: 3.612 × 10^5^	-	100 Hz	20/50	6.79	unstable with time	[82]
VA-MWNT *	40–90		6.6 Ω/RH%	-	3.3/71	1.5%	1800 s	[83]
CNF/CNT *	29–95	ΔI/I%: 87.3	-	-	322/442	5.9%	2 months	[14]
TF(SnO_2_-R) *	30–90	*Z_L_*/*Z_H_*_:_ 160	406.8 kΩ/RH%	-	4/6	3.7	-	[16]
LrGO/MWNT	11–97	ΔZ/Z%: 10.1 × 10^5^ΔC/C%: 6.9 × 10^4^	0.35 MΩ/RH%798 pF/%RH	@100 Hz@ 40 Hz	61 ms/2.3 s	3.1	3 years	This work

* LrGO: laser-reduced GO; LIG: laser-induced carbon; BP: black phosphorene; HNTs: halloysite nanotubes; CNC: nanocellulose; VA-MWNT: vertically aligned MWNT, CNF/CNT: cellulose nanofiber/carbon nanotube, TF(SnO_2_-R): thick film KIT-5-mediated synthesized SnO_2_ replica.

#### 3.2.5. Analysis of Impedance Spectroscopy and Humidity Response

**Figure 9:**
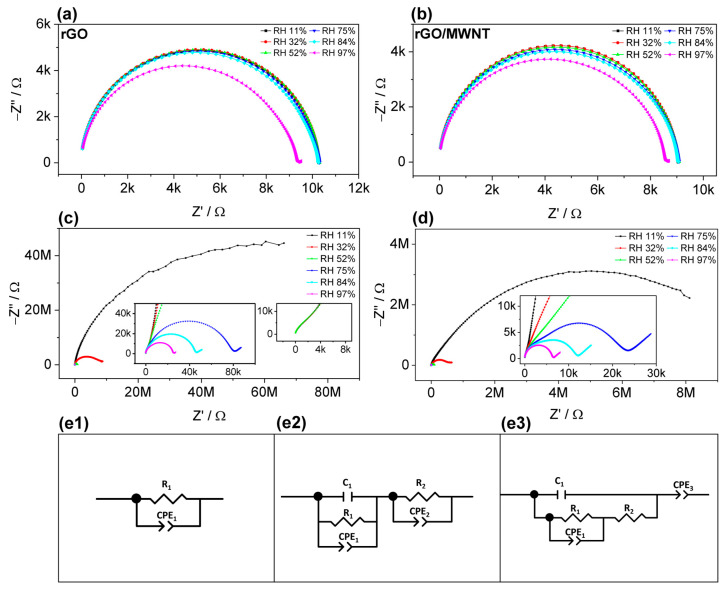
Nyquist representation of impedance for (**a**) rGO rectangular films, (**b**) rGO/MWNT, (**c**) rGO-GO-rGO IDE, and (**d**) rGO/MWNT-GO/MWNT-rGO/MWNT IDE. Equivalent circuits for (**e1**) rGO and rGO/MWNT films, (**e2**) IDE structure at RH 11–52%, (**e3**) IDE structure at RH 75–97%.

The impedance spectroscopy was performed in the frequency range of 40 Hz to 110 MHz. In Figure 9, complex impedance spectra (CIS) in the Nyquist plot are shown for the sensors mentioned above, the rectangular shape (rGO and rGO/MWNT sensors), and the GO IDE and GO/MWNT IDE sensors. 

Structured rectangular sensor films have an initial impedance of several kΩ. They have semi-circle shapes overlapping and almost fixed real parts for most of the tested humidity points except a significant decrease at 97% RH, as discussed in Section 3.2.1. In general, they presented resistive behavior in a wide range of frequencies, as shown in the Bode plot (Figure S4). The semi-circle is slightly depressed at frequencies around 7 MHz (Figure 9a). The complex impedance can be represented as a charge transfer resistance (denoted as R_1_) corresponding to rGO resistance in parallel with a constant phase element (CPE), denoted as CPE_1_, for the interface between the electrode and rGO reflecting the surface irregularities and the remaining unreduced GO areas [84], Figure 9(e1). and its impedance described by Equation (4) [85],

(4) $$Z_{CPE}=\frac{1}{Q{\left(i\omega \right)}^{n}}$$

where *n* is a dispersion parameter and *Q* in F/s^(1−n)^; as can be seen, the resistance varies little with RH, having a significant drop only at the highest RH, and the resistance is around 10 kΩ. The same explanation can be applied to rGO/MWNT material (Figure 9b). This CIS behavior was found for thermally based rGO as demonstrated in a previous report [65], similar to behavior to partially rGO due to remaining GO layers.

For the IDE structure of both GO and GO/MWNT (Figure 9c,d), high sensitivity is observed with a drastic depression of the semi-circle in the RH range of 11–52%. However, the behavior is rather complex, suggesting that the equivalent circuit evolves as humidity increases. The humidity affects both reduced and unreduced areas of the sensors. Therefore, the impedance spectra reflect the combination of both materials systems, which behave differently.

Most GO-based humidity sensors show evolving equivalent circuit models as CPE − CPE/R_f_ − CPE/R_f_ + CPE, corresponding to low-medium-high humidity, which may be suitable for GO with metallic electrodes [33,69,76]. Another equivalent circuit model (R_f_/C_f_ − C_f_/(R_f_ + *Z_w_*)) uses the Warburg diffusion element [72,77,86]. However, it should be noted that using LCR meters, as in many previous studies, cannot reveal the whole impedance spectra due to the limit of the frequency range. Therefore, the whole impedance spectra range at higher humidities with full semi-circle cannot be obtained [41,42,68,71,75], and it is difficult to distinguish more representative circuit elements in a limited frequency range and hence establish a suitable model. For example, the frequency associated with the maximum of the semi-circle of rGO is at around 7 MHz (Figure 9a and Figure S4), which needs frequencies at several 10 MHz to obtain the full spectra (complete semi-circle). In the case of all-graphene sensors based on laser rGO-GO-rGO structure have utilized the same equivalent circuit ((R_f_ + *Z_w_*)/C_f_), where *Z_w_* represents the water diffusion impedance, to interpret the humidity sensing mechanism [43,76]. However, this model cannot be considered as it underestimates the rGO-GO heterojunction and the contribution of rGO, which can contribute significantly, depending on the degree of reduction. A different model was proposed in [41] by simulation that fits best for the RH value of 33%, comprising a heterojunction model with Warburg element. Probably, using low frequencies starting from 0.4 Hz to 4000 Hz has enabled the authors to reveal a dispersion line, which is not the case at the 33% RH level in most of the reported papers. In the high frequency, the semi-circle is incomplete, and there was no further semi-circle observable from the spectra belonging to rGO electrodes.

Here, the complex impedance spectra show two partial semi-circles arcs. The small one is visible in the high-frequency range at ~7 MHz, Figure 5d inset. This is where the characteristic frequency of the rGO and rGO/MWNT semi-circle maximum is located, as mentioned above. Therefore, we suggest different models for different humidity ranges. For IDE fingers of rGO or rGO/MWNT, it can be represented as CPE/R, as discussed earlier. For GO or GO/MWNT active channels at low humidities, it can be represented by a CPE element [29]. In addition, at the interface between rGO-GO, it can be modeled as double-layer capacitance and charge transfer resistance C_1_/R_1_ as the applied potential (50 mV) is slightly greater than the open-circuit potential [85,87]. Thus, the resulting circuit corresponding to GO or GO/MWNT is CPE_1_/R_1_, as shown in Figure 9(e2) and marked with (1). Elements marked with (2) correspond to the IDE structure, and the values of the charge transfer resistance found by the circuit fitting were similar to that of rGO and rGO/MWNT layers (R_2_/CPE_2_). This applies to the humidity range of 11–52%, where fewer water ions are available.

At higher humidities, 75–97% RH, by the increase in water content, the equivalent circuit can be represented as a heterostructure-like circuit model for electrode interface in corrosion or solar cells with several stacked materials layers [88]. In our case, it is an in-plane heterojunction between the IDE structure and the non-reduced GO (GO/MWNT) layer of rGO-GO or rGO/MWNT-GO/MWNT. In Figure 9e, the capacitance (C_1_) corresponds to the GO-rGO double-layer capacitance, the resistance (R_2_) is the contact resistance of the rGO fingers, and C_1_/R_1_/CPE_1_ is the total for GO + rGO or GO/MWNT + rGO/MWNT, which likely assembles the complex impedance of the interfaces with different reduction levels along the sensing layer depth profile at the contact between the reduced and non-reduced part of the sensor. Thus, we suggest that such an evolution of the equivalent circuit is due to the gradient of the reduction degree in the IDE structure and penetration of water molecules in the deeper parts of the sensing layers. These water molecules adsorbed through the oxygen-containing functional group will be ionized into H_3_O^+^ under the action of electric field force, which reduces the sensor’s resistance. The proton hopping transport is easily formed in the water layers formed via Grotthuss reaction (H_2_O + H_3_O^+^ → H_3_O^+^ + H_2_O) [86]. Additionally, with the increase in the water amount adsorbed by the material, the dielectric constant also increases, which increases the capacitance and decreases the capacitive resistance of the material. The diffusion of water molecules in the sensing layer also induced a diffusion-like tail in impedance spectra, visible for the RH range of 75–97%. The fitting results for GO/MWNT IDE sensor are shown in Table S2 and Figure S7a–c. Figure S7d–f shows the characteristics of the equivalent circuit parameters obtained from the equivalent circuit fitting of the CIS versus RH%. All plotted parameters can be related to the RH variation. The parameter n_1_ reflects more capacitive behavior, while n3 reflects ionic behavior with a tendency to resistive behavior with an RH% increase [89]. While C_1_ and CPE_2_, Table S2, seem to have no significant relevance to humidity, R_2_ corresponds to rGO resistance increase with humidity with the rise of humidity [90]. 

## 4. Conclusions

In this work, direct laser-structured GO-based humidity sensors were characterized. GO moisture-sensitive properties of the GO-based humidity sensor were additionally enhanced, using MWCNT to form GO/MWNT sensing layers. Based on the obtained results, MWNTs interact with GO to form a stable composite, enhancing the adsorption properties and reducing the high impedance of GO at low humidity, making the impedance measurement easier and more reliable. GO/MWNT-based sensor has a humidity response in the tested range of RH 10% to 97%. At the working frequency of 1 kHz, its sensitivity is 4.6 × 10^4^%, while the range of impedance is from 7.3 kΩ to 3.2 MΩ. Moreover, it also has an excellent performance in terms of hysteresis, repeatability, and several years of stability. The measured response/recovery times in the humidity chamber were 10.7 s and 9.3 s. However, by respiration test followed by dry air dehumidification, they measured as low as 61 ms and 2.3 s. By direct laser scribing, a humidity-insensitive film can be produced where no additional coating is required, which is beneficial for other sensor applications. GO/MWNT material has potential in the future of the humidity sensors field. This expectation is not only based on excellent sensing characteristics but also on the exceptional stability of such sensors.

## Data Availability

The data are available upon request to the corresponding author.

## Supplementary Materials

The following supporting information can be downloaded at: https://www.mdpi.com/article/10.3390/nano13091473/s1, Figure S1: GO IDE sensors with different fingers gaps (a) digital design (10.5 × 12.2 mm), (b) camera image of the sensor, and (c) optical microscope image of the IDE structure; Figure S2: (a) Photograph of the humidity measurement procedure (using saturated salts and impedance analyzer) (b) two gas flow humidity measurement setup and (c) humidity and temperature measurements inside the chamber in (b); Figure S3: SEM images and corresponding C and O EDX maps of (%) GO, (b) reduced GO, (c) GO/MWCNT composites, and (d) reduced GO/MWCNT composite; Figure S4: Bode–Bode plot corresponding to Figure 9 in the manuscript; (a) rGO rectangular films, (b) rGO/MWNT; Figure S5: Bode-Bode plot corresponding to Figure 9 in the manuscript; (a) rGO-GO-rGO IDE and (b) rGO/MWNT-GO/MWNT-rGO/MWNT IDE; Figure S6: Stability of the (a) rGO and (b) rGO/MWNT films inside different humidity points, sensors are from different production batch; Figure S7 Fitting of GO/MWNT IDE sensor: (a) Nyquist plot, Bode plot of (b) impedance and (c) phase values of (d) R_1_, (e) Q_1_, n_1_, and (f) Q_3_, n_3_ parameters obtained from the equivalent circuits fitting versus RH%; Table S1: IDE sensor dimension specification; Table S2: Equivalent circuit parameter values for rGO/MWNT IDE sensor at different humidities. The corresponding fittings are shown in Figure S6; Video S1: GO/MWNT sensor and actuation demonstration.
