# Supplementary material for: Ultra-Sensitive and Fast Humidity Sensors Based on Direct Laser-Scribed Graphene Oxide/Carbon Nanotubes Composites
Source: Nanomaterials (Basel). 2023 Apr 26;13(9):1473. doi: 10.3390/nano13091473 (PMC10180099; doi:10.3390/nano13091473)
Supplement: Supplementary file 1 [file nanomaterials-13-01473-s001.zip › Supp_info_LrGO-CNT based humidity sensor-PR.zip!/Supp_info_LrGO-CNT based humidity sensor-PR.pdf]

Supplemetry Information

Ultra-Sensitive and Fast Humidity Sensors Based on Direct Laser-Scribed Graphene Oxide/Carbon Nanotubes Composites

Ammar Al-Hamry [1,*], Tianqi Lu [1], Haoran Chen [1], Anurag Adiraju [1], Salem Nasraoui [1], Amina Brahem [1], Danica Bajuk-Bogdanović [2], Saddam Weheabby [1], Igor A. Pašti [2] and Olfa Kanoun [1,*]

1 Measurement and Sensor Technology, Department of Electrical Engineering and Information Technology, Chemnitz University of Technology, 09107 Chemnitz, Germany; tianqi.lu@etit.tu-chemnitz.de (T.L.); adiraju.anurag@etit.tu-chemnitz.de (A.A.); salem.nasraoui@etit.tu-chemnitz.de (S.N.); amina.brahem@etit.tu-chemnitz.de (A.B.); saddam.weheabby@etit.tu-chemnitz.de (S.W.)
2 University of Belgrade—Faculty of Physical Chemistry, Studentski trg 12–16, 11158 Belgrade, Serbia; danabb@ffh.bg.ac.rs (D.B.-B.); igor@ffh.bg.ac.rs (I.A.P.)
* Correspondence: ammar.al-hamry@etit.tu-chemnitz.de (A.A.-H.); olfa.kanoun@etit.tu-chemnitz.de (O.K.)

Figure S1 shows the detail of the IDE based sensors, S1-S4, which have a whole geometry of 10.5 × 12.2 mm. The differences are in the gap between fingers, 300, 400, 500 and 600 μm and the number of fingers, 7, 9, 11 and 14, respectively. Laser writing was done 6 times for all the structures. For all sensor the finger width was digitally 600 μm and after laser writing it results in 425.37±22.95 μm. In Table S1, a comparison of gap width before and after laser writing for sensors S1-S4.

Table S1 IDE sensor dimension specification.

Sensor No.	Number of fingers	Gap width (μm)	
		Digital design	After laser writing
S1	7	600	442.21 ± 26.66
S2	9	500	389.45 ± 27.42
S3	11	400	238.61 ± 2.91
S4	14	300	179.04 ± 19.67

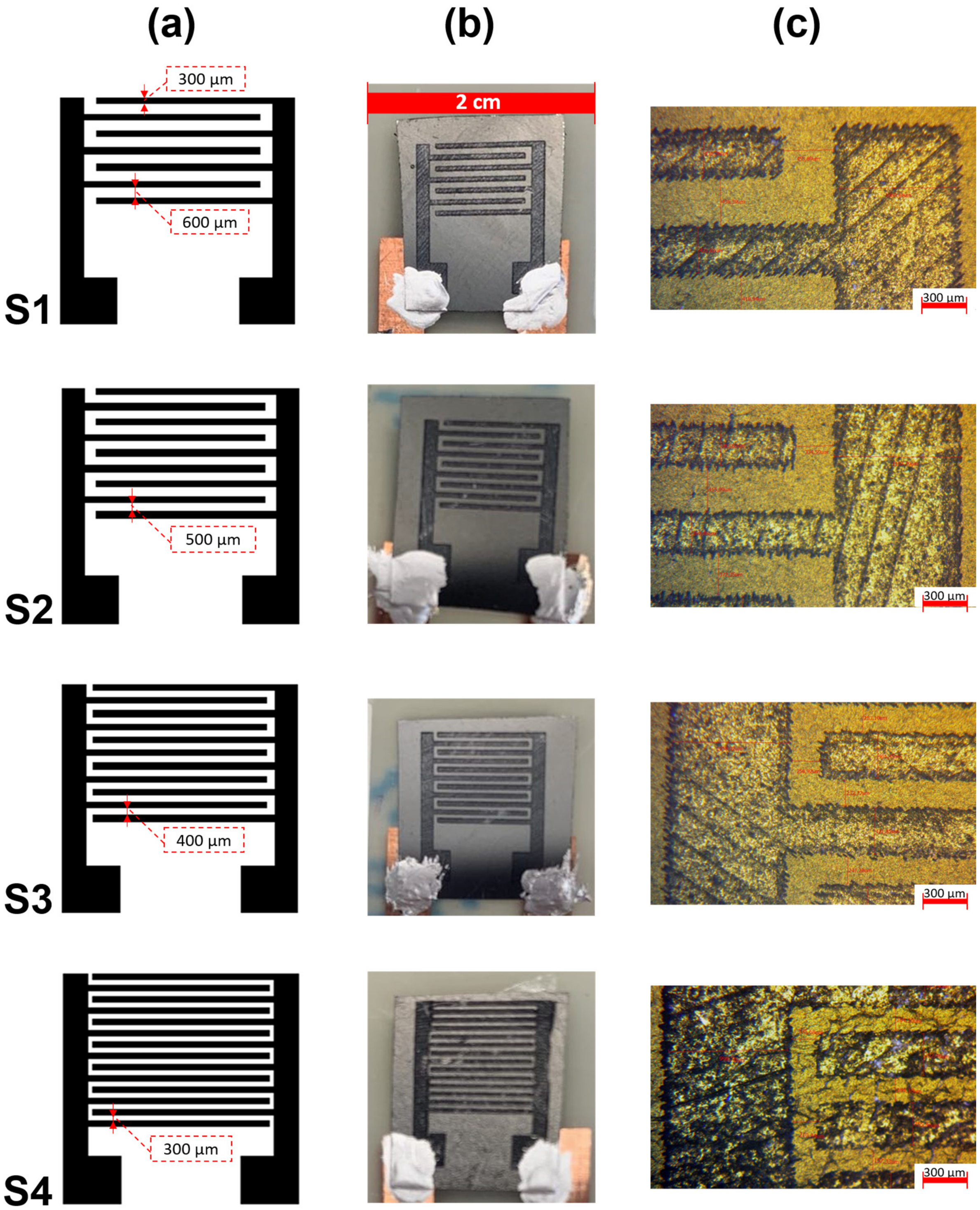

Figure S1 GO IDE sensors with different fingers gaps: (a) digital design (10.5 × 12.2 mm), (b) camera image of the sensor and (c) optical microscope image of the IDE structure.

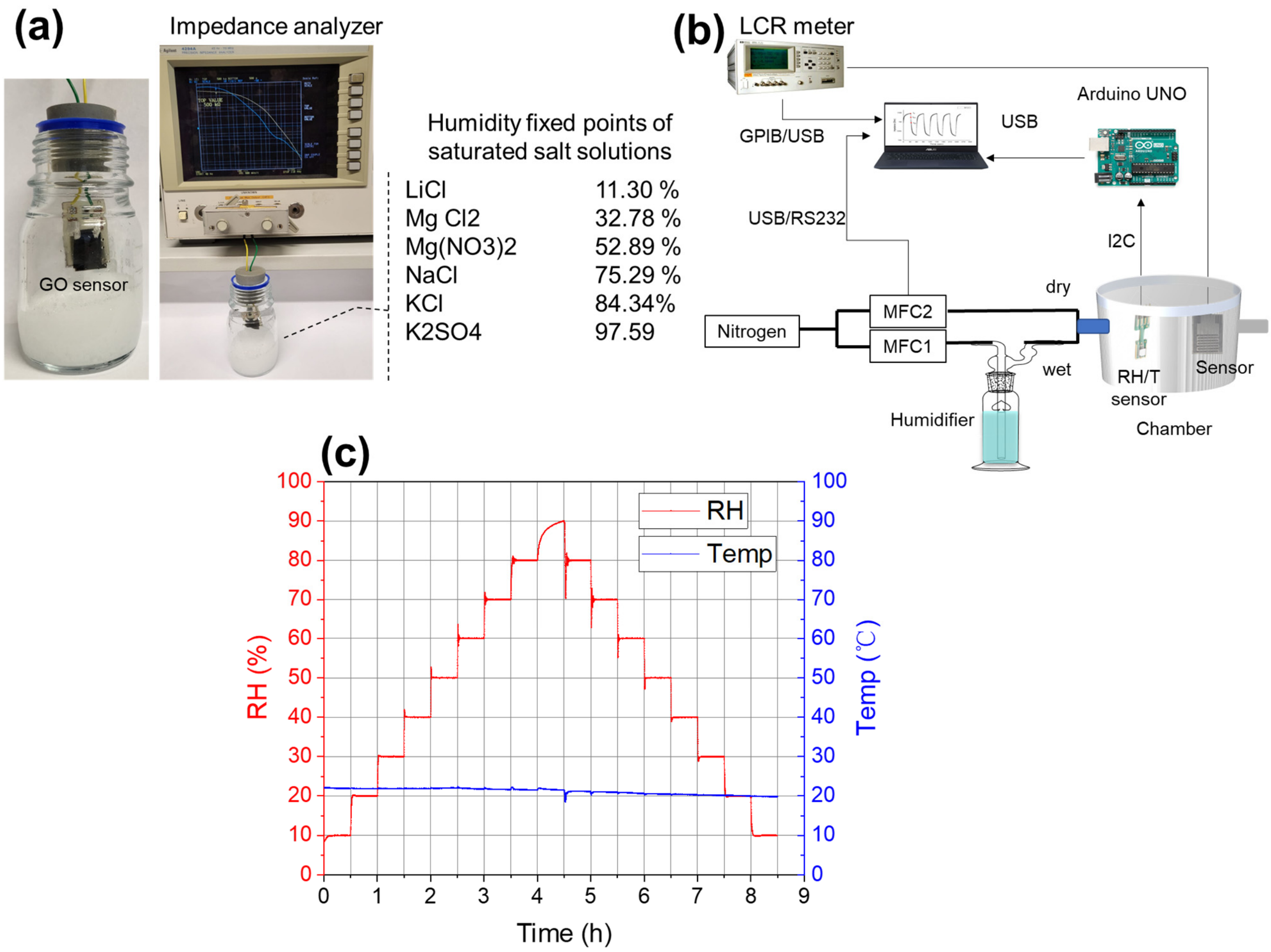

Figure S2 (a) Photograph of the humidity measurement procedure (using saturated salts and impedance analyzer) (b) two gas flow humidity measurement setup and (c) humidity and temperature measurements inside the chamber in (b).

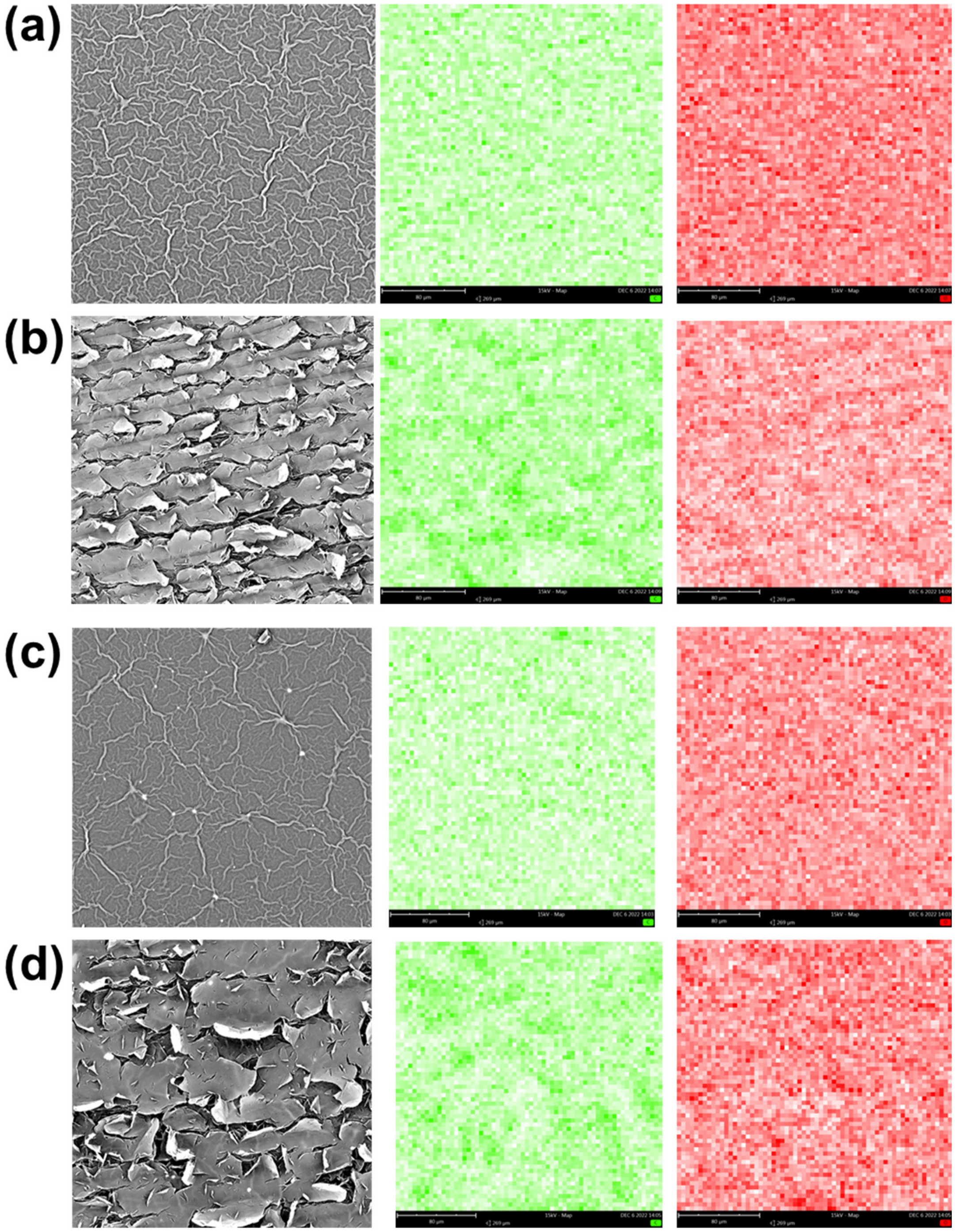

Figure S3 SEM images and corresponding C and O EDX maps of (a) GO, (b) reduced GO, (c) GO/MWCNT composites, and (d) reduced GO/MWCNT composite.

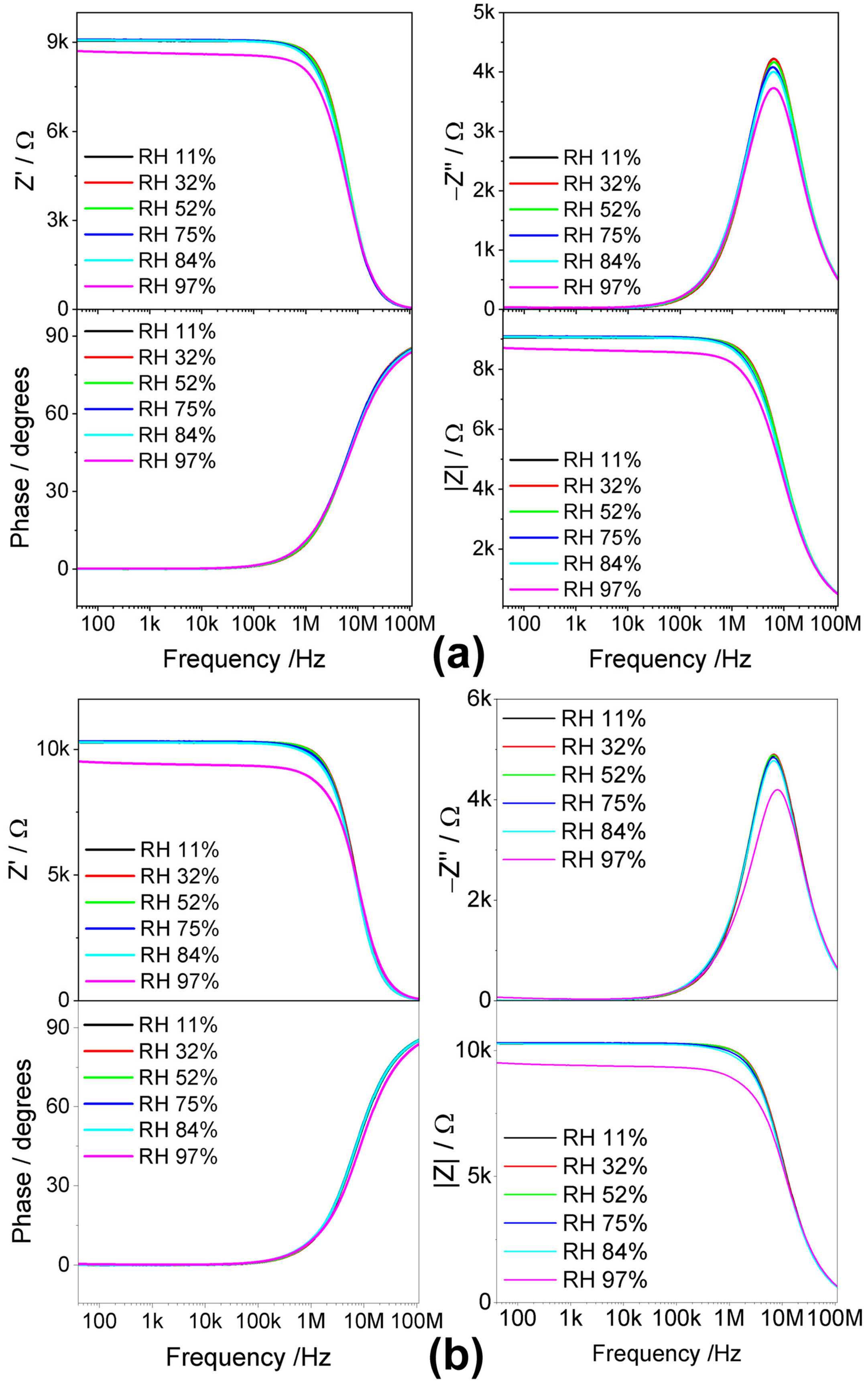

Figure S4 Bode-Bode plot corresponding to Figure 9 in the manuscript; (a) rGO rectangular films, (b) rGO/MWNT.

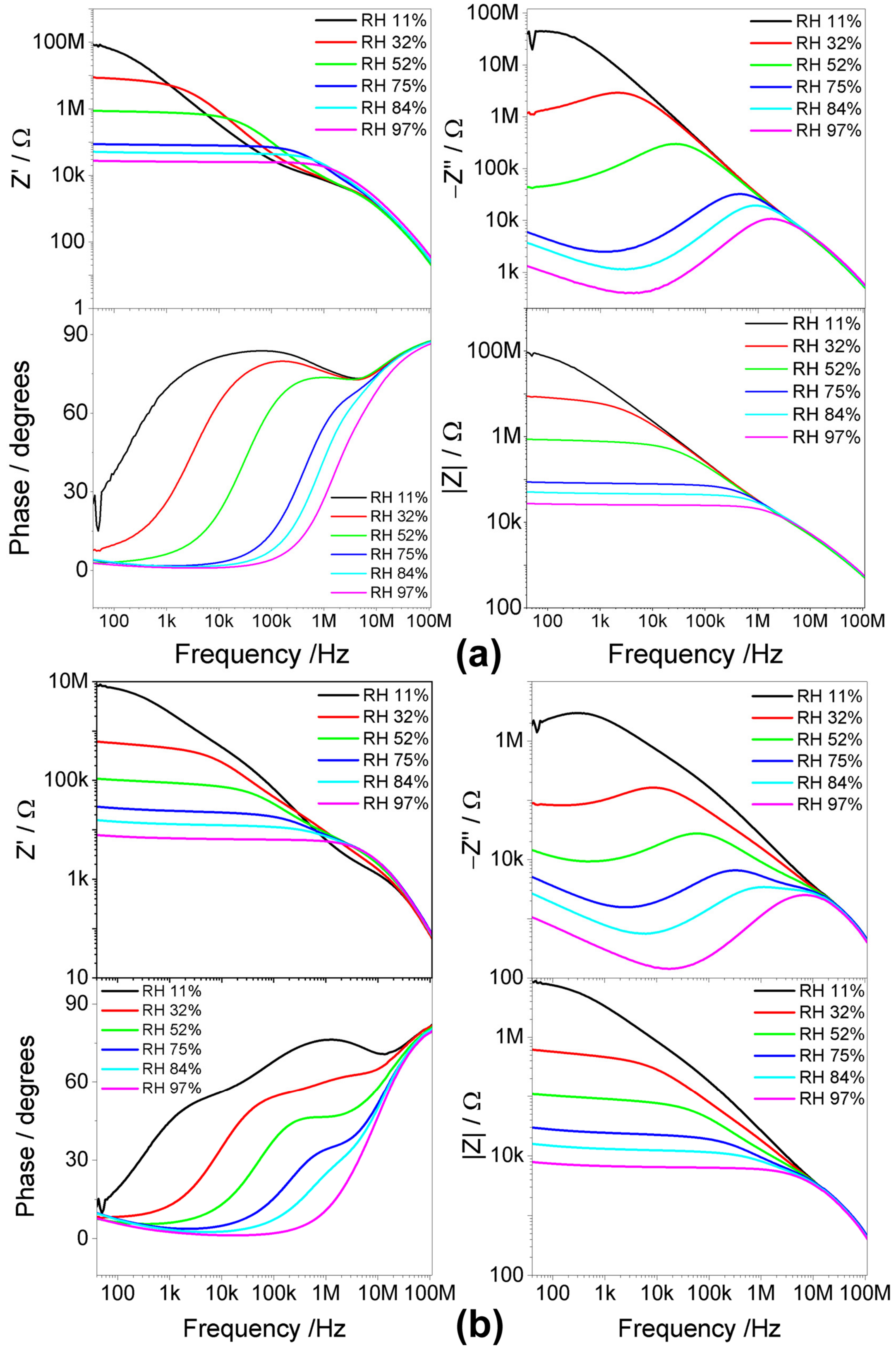

Figure S5 Bode-Bode plot corresponding to Figure 9 in the manuscript; (a) rGO-GO-rGO IDE and (b) rGO/MWNT-GO/MWNT-rGO/MWNT IDE.

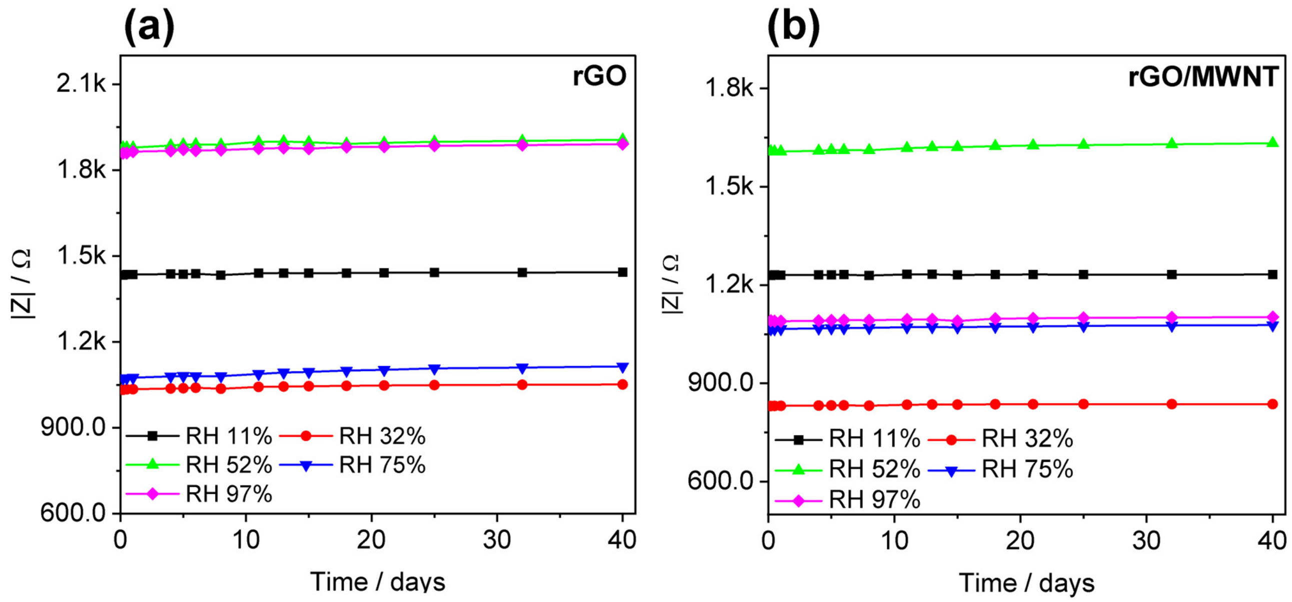

Figure S6 Stability of the (a) rGO and (b) rGO/MWNT films inside different humidity points, sensors are from different production batch.

Table S2 Equivalent circuit parameter values for rGO/MWNT IDE sensor at different humidities. The corresponding fittings are shown in Figure S6.

	RH 11%	RH 32%	RH 52%		RH 75%	RH 84%	RH 97%
	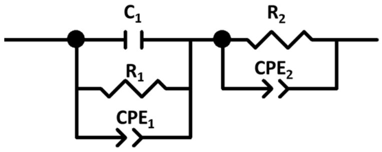				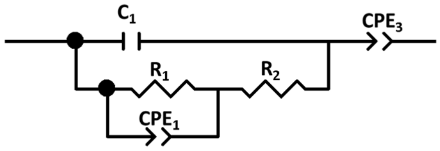		
C_1 (pF)	4.42±0.57%	5.56±0.62%	3.14±1.81%	C_1 (pF)	2.32±0.41%	2.42±0.29%	2.27±0.25%
R_1(kΩ)	5830.1±0.37 %	6316.1±1.36%	1282.9±0.32%	R_1(kΩ)	100.1±0.38%	34.77±0.36%	5.77±8.78%
Q_1 (nF s^{n-1})	1.38±1.94%	14.31±1.96%	9.25±1.36%	Q_1 (nF)$^{n-1}$	4.36±2.81%	2.89±3.48%	0.54±1.11%
n_1	0.56±0.34%	0.42±0.43%	0.52±0.24%	n_1	0.62±0.34%	0.66±0.39%	0.76±0.83%
R_2 (kΩ)	3.82±1.41%	5.33±1.61%	2.77±3.07%	R_2 (kΩ)	7.97±0.86%	7.73±0.63%	5.93±0.83%
Q_2 (pF s^{n-1}	18.17±5.75%	23.09±6.55%	9.32±7.63%	Q_3 (μF)$^{n-1}$	6.83±4.26%	12.9±2.25%	50.74±2.77%
n_2	0.92±0.33%	0.9±0.38%	0.97±0.47%	n_3	0.38±1.01%	0.36±0.84%	0.34±1.49%

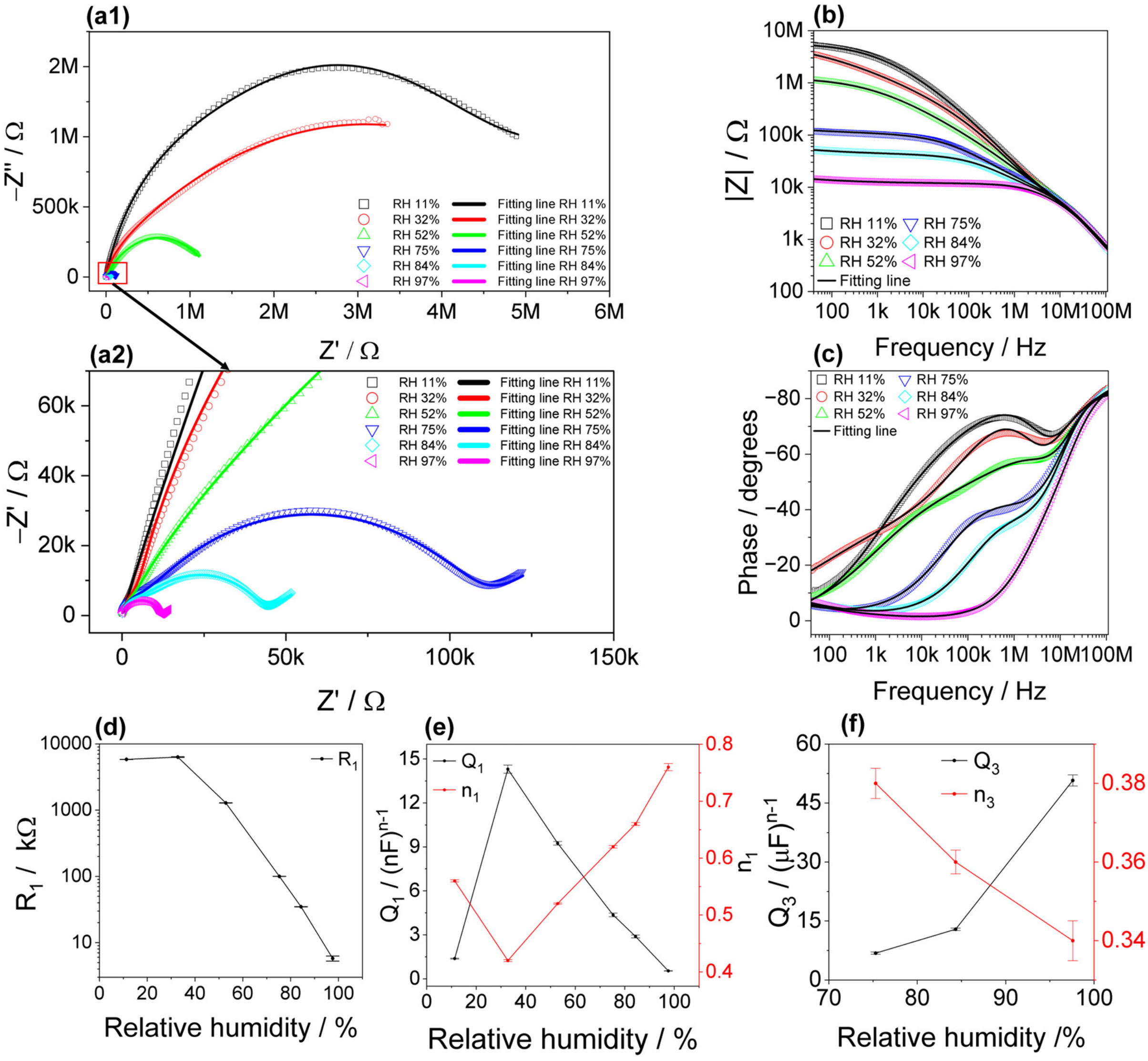

Figure S7 Fitting of GO/MWNT IDE sensor: (a) Nyquist plot, Bode plot of (b) impedance and (c) phase. values of (d) R_1, (e) Q_1-n_1, and (f) Q_3-n_3 parameters obtained from the equivalent circuits fitting versus RH%.
